# Burden of Multidrug-Resistant Uropathogens and Their Antimicrobial Susceptibility Among Hospitalized Patients: A Single-Center Retrospective Analysis

**DOI:** 10.7759/cureus.112449

**Published:** 2026-07-11

**Authors:** Divita Rohatgi, Harish Moorjani

**Affiliations:** 1 Medicine, Hamdard Institute of Medical Sciences and Research, New Delhi, IND; 2 Infectious Diseases, Phelps Hospital, Sleepy Hollow, USA

**Keywords:** antimicrobial stewardship practice, multidrug-resistant organism, single-center retrospective study, urinary tract infections, uropathogens

## Abstract

Background

Urinary tract infections (UTIs) represent a major global health challenge, with the rise of multidrug-resistant (MDR) uropathogens significantly complicating empiric treatment and increasing medical costs. The objective of this study was to determine the local burden of MDR UTIs and the patient characteristics associated with it, characterize the causative pathogens, and describe their antimicrobial susceptibility patterns in a community hospital setting.

Methods

A retrospective cross-sectional study was conducted at Phelps Hospital in Sleepy Hollow, NY, USA, utilizing 392 urine samples collected from inpatients between February and May 2026. MDR status was defined as acquired non-susceptibility to at least one agent in three or more antimicrobial categories. Bacterial isolates were identified using automated methods, followed by antimicrobial susceptibility testing interpreted according to Clinical and Laboratory Standards Institute (CLSI) 2022 guidelines.

Results

Among 286 symptomatic patients, 70 (70/286; 24.47%) had MDR UTIs. *Escherichia coli* was the predominant pathogen (48/70 samples; 68.6%), of which 47.91% (23/48 samples that grew *Escherichia coli*) were extended-spectrum beta-lactamase (ESBL)-producing. A significant finding was that among the MDR isolates that grew *Enterococcus*, all were vancomycin-resistant. The most common clinical characteristics associated with MDR infection were prior antibiotic use within six months (42/70; 60%) and hospitalization within the previous year (41/70; 58.7%). Susceptibility data revealed high activity of carbapenems and tigecycline against the majority of MDR gram-negative isolates in this cohort. Conversely, high resistance rates were observed for fluoroquinolones and trimethoprim-sulfamethoxazole (TMP-SMZ), making them unreliable for empiric use in this population.

Conclusion

The high prevalence of ESBL-producing *Enterobacterales* and vancomycin-resistant enterococci (VRE) in this cohort emphasizes the critical need for routine urine culture and susceptibility testing to guide effective therapy. Implementing antibiotic stewardship programs and utilizing local resistance data are essential to prevent the further emergence of resistance and to preserve the efficacy of last-resort antibiotics.

## Introduction

Urinary tract infections (UTIs) are common bacterial infections that affect millions of people worldwide each year, causing significant morbidity and incurring billions of dollars in annual costs [1,2]. In general, UTIs are classified by site of infection (cystitis, urethritis, or pyelonephritis); severity (uncomplicated or complicated); or as primary or recurrent [3]. UTIs are caused by a wide range of organisms, with bacteria accounting for >95% of cases [4]. The bacterial pathogens involved in UTIs are mainly Gram-negative, including *Escherichia coli*, *Klebsiella pneumoniae*, *Pseudomonas aeruginosa*, *Citrobacter*, *Enterobacter*, and *Proteus *species, with *Escherichia coli* being the most common [5]. Among Gram-positive bacteria, *Staphylococcus aureus*, *Staphylococcus saprophyticus*, and *Enterococcus *species are commonly responsible for UTIs [6]. The relative frequency of uropathogens also depends on patient age and sex, catheterization, previous exposure to antimicrobials, and the presence of comorbid conditions such as diabetes mellitus [7-9].

The frequent prescription of broad-spectrum antibiotics for empiric treatment has led to resistance among common pathogens [10]. Over the past few years, the prevalence of multidrug-resistant (MDR) uropathogens has increased in both community and hospitalized patients [11]. According to the consensus proposal by Magiorakos et al., developed through a joint initiative of the European Centre for Disease Prevention and Control (ECDC) and the Centers for Disease Control and Prevention (CDC), MDR organisms are defined as those demonstrating acquired non-susceptibility to at least one agent in three or more antimicrobial categories [12]. This limits the antibiotic options available for treating these pathogens. Antibiotic susceptibility patterns vary across hospitals and geographical locations [13]. The Infectious Disease Society of America (IDSA) recommends regional surveillance to monitor variations in antibiotic susceptibility patterns [14]. The emergence of MDR pathogens makes it imperative to identify the causative agent of UTIs and determine its antibiotic susceptibility to successfully treat UTIs and prevent the indiscriminate use of antibiotics [15]. This study aimed to determine the local burden of MDR UTIs, the patient characteristics associated with them, their causative pathogens, and the antimicrobial susceptibility pattern among individuals with suspected UTIs in a community hospital setting.

## Materials and methods

Ethics statement

This study was reviewed and approved by the Northwell Health Institutional Review Board (IRB). The study involved retrospective analysis of fully anonymized secondary data from laboratory records of urine samples submitted for diagnostic purposes. As the data were de-identified and no intervention or direct patient interactions occurred, the IRB granted a waiver of informed consent. All procedures adhered to the institutional ethical guidelines.

Study design

A retrospective cross-sectional study was conducted using an anonymized secondary dataset comprising urine culture and sensitivity test results collected between February and May 2026 at Phelps Hospital, Sleepy Hollow, a community-based hospital in New York State, USA. The microbiology laboratory database was reviewed to identify all patients with positive urine cultures in the designated study period (February-May, 2026), yielding 392 patients with culture-positive urine samples. For patients with multiple positive urine cultures during the study period, only the first episode was included in the analysis to avoid duplicate patient entries. These cases constituted the initial study population and were screened for eligibility. Medical records were subsequently reviewed to determine whether patients had clinical signs and symptoms consistent with a UTI during their hospitalization. Patients with asymptomatic bacteriuria or urine cultures interpreted as representing urinary tract colonization rather than true infection were excluded, which yielded 286 patients with symptomatic, culture-confirmed urinary tract infections, who were included in the final analysis. As this was a retrospective observational study, no formal prior sample size calculation was performed. The present study did not involve direct contact with the patients. 

Diagnostic criteria

The diagnostic criteria for UTI were clinical symptoms such as dysuria, frequency, suprapubic pain, fever, chills, and lumbar pain, in addition to a leukocyte count of ≥10/mm³ in non-centrifuged urine and/or pathogenic bacterial growth of ≥10⁵ in bacterial culture. MDR UTI was defined as a UTI caused by a microbe demonstrating acquired non-susceptibility to at least one agent in three or more antimicrobial categories.

Sample collection and processing

At first, midstream, clean-catch urine samples were obtained from each patient using wide-mouth, screw-capped, sterile containers, following standard precautions to prevent contamination. Each sample had a minimum volume of 5 mL and was transported to the microbiology laboratory within 30 minutes of collection. The samples were inoculated onto sheep blood agar and MacConkey agar and incubated aerobically at 37 °C for 16-18 hours.

Laboratory analysis

Bacterial Isolation, Identification, and Antimicrobial Susceptibility Testing

Definitive species-level identification was performed using matrix-assisted laser desorption/ionization time-of-flight mass spectrometry (MALDI-TOF MS) in accordance with standard laboratory protocols. Antimicrobial susceptibility testing was performed using the VITEK® 2 automated system (bioMérieux SA, Marcy-l'Étoile, France), and the results were interpreted according to the 2022 Clinical and Laboratory Standards Institute (CLSI) guidelines [16]. Antimicrobial susceptibility testing was performed using a broad range of antibiotics, categorized by class: aminoglycosides (amikacin, gentamicin, tobramycin), beta-lactam/beta-lactamase inhibitor combinations (amoxicillin-clavulanate, piperacillin-tazobactam), penicillins (ampicillin, penicillin), cephalosporins (cefazolin, cefepime, cefixime, cefotaxime, cefoxitin, ceftaroline, ceftazidime-avibactam, ceftazidime, ceftriaxone, cefuroxime), carbapenems (imipenem, meropenem), quinolones (nalidixic acid), fluoroquinolones (ciprofloxacin, levofloxacin), macrolides (azithromycin, erythromycin), tetracyclines (doxycycline, tetracycline, tigecycline), glycopeptides (vancomycin), sulfonamides (trimethoprim-sulfamethoxazole (TMP-SMZ)), phenicols (chloramphenicol), lincosamides (clindamycin), polypeptides (colistin), fosfomycin, and oxazolidinones (linezolid). Due to the limited availability of certain antibiotic discs, uniform drug sensitivity testing could not be performed for all isolates.

## Results

**Figure 1:**
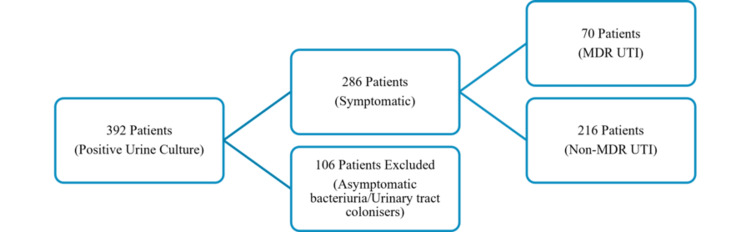
Patient selection process in the study MDR UTI: multidrug-resistant urinary tract infection

During the study period, 392 patients had positive urine cultures; 106 with asymptomatic bacteriuria or urinary tract colonization were excluded. Of the remaining 286 patients, 70 had MDR UTI (70/286; 24.47%). Figure 1 describes the patient selection process in the study.

**Figure 2:**
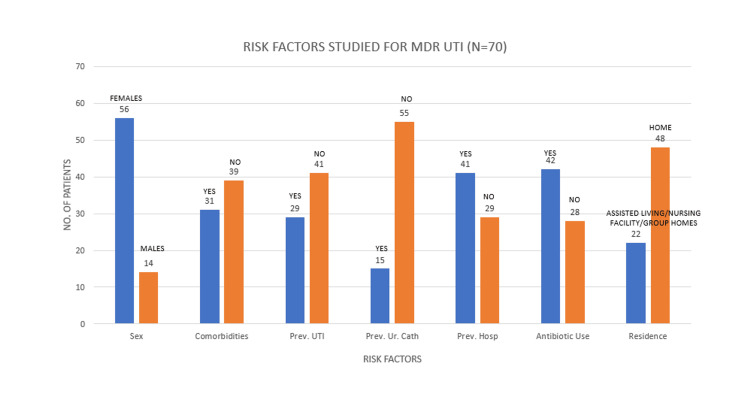
Demographic and clinical characteristics studied in patients with MDR UTIs (N = 70) MDR UTIs: multidrug-resistant urinary tract infections; Prev.: previous; Cath: catheterization; Hosp: hospitalization

**Figure 3:**
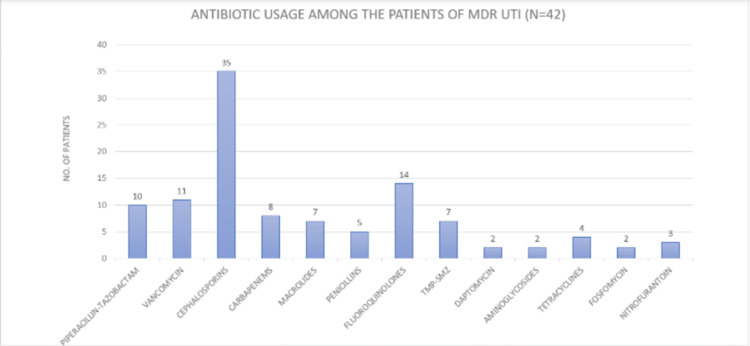
Pattern of prior antibiotic usage among patients with MDR UTIs (N = 42); Of the 70 patients with MDR UTIs, 42 had prior antibiotic use. MDR UTI: multidrug-resistant urinary tract infection; TMP-SMZ: trimethoprim-sulfamethoxazole

Of the 70 patients with MDR UTIs, 56 were females (80%). A total of 31 of 70 patients (44.28%) had an associated comorbid condition, such as type 2 diabetes mellitus, chronic kidney disease, immunosuppression, or neurogenic bladder. Of the 70 patients, a history of UTIs in the past 12 months was recorded in 29 patients (41.42%), while previous catheterization within the same period was documented in 15 patients (21.33%). Hospitalization in the past year was noted in 41 patients (58.57%). A history of antibiotic use during the last six months was present in 42 patients (42/70; 60%). Among these 42 patients, antibiotics used included piperacillin-tazobactam, vancomycin, cephalosporins, carbapenems, macrolides, penicillins, fluoroquinolones, TMP-SMZ, daptomycin, aminoglycosides, tetracyclines, fosfomycin, and nitrofurantoin, which were noted in 10 (23.80%), 11 (26.19%), 35 (83.33%), eight (19.04%), seven (16.66%), five (11.90%), 14 (33.33%), seven (16.66%), two (4.76%), two (4.76%), four (4.76%), two (4.76%), and three (7.14%) patients, respectively. Additionally, 22 of 70 patients (31.42%) lived in assisted living facilities, nursing homes, or group homes. Figures 2-3 summarize the clinical characteristics of patients with MDR UTI and the distribution of prior antibiotic use among them, respectively.

**Figure 4:**
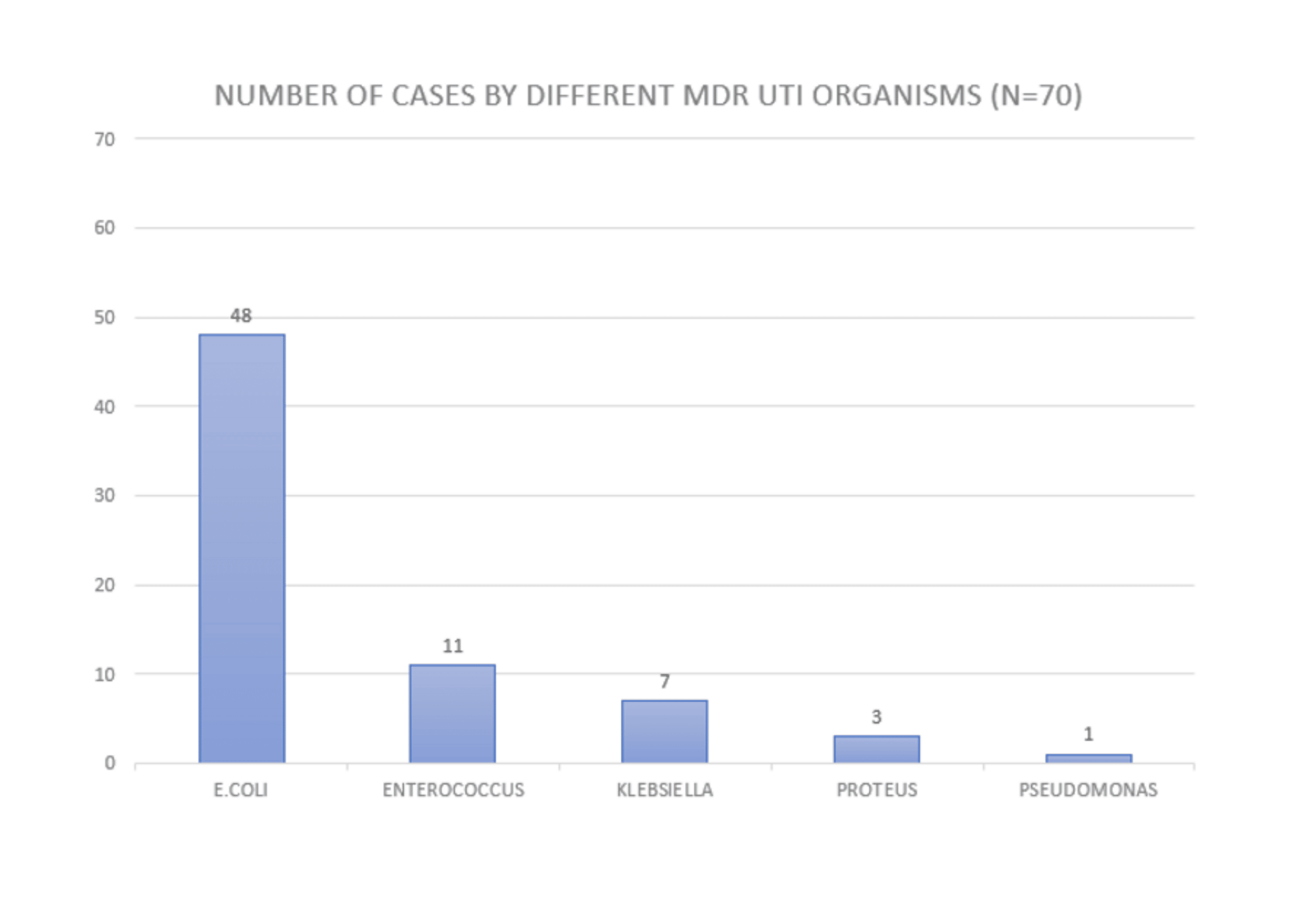
Distribution of MDR UTI cases by causative organisms (N = 70) MDR UTI: multidrug-resistant urinary tract infection; *E. coli*: *Escherichia coli*

**Figure 5:**
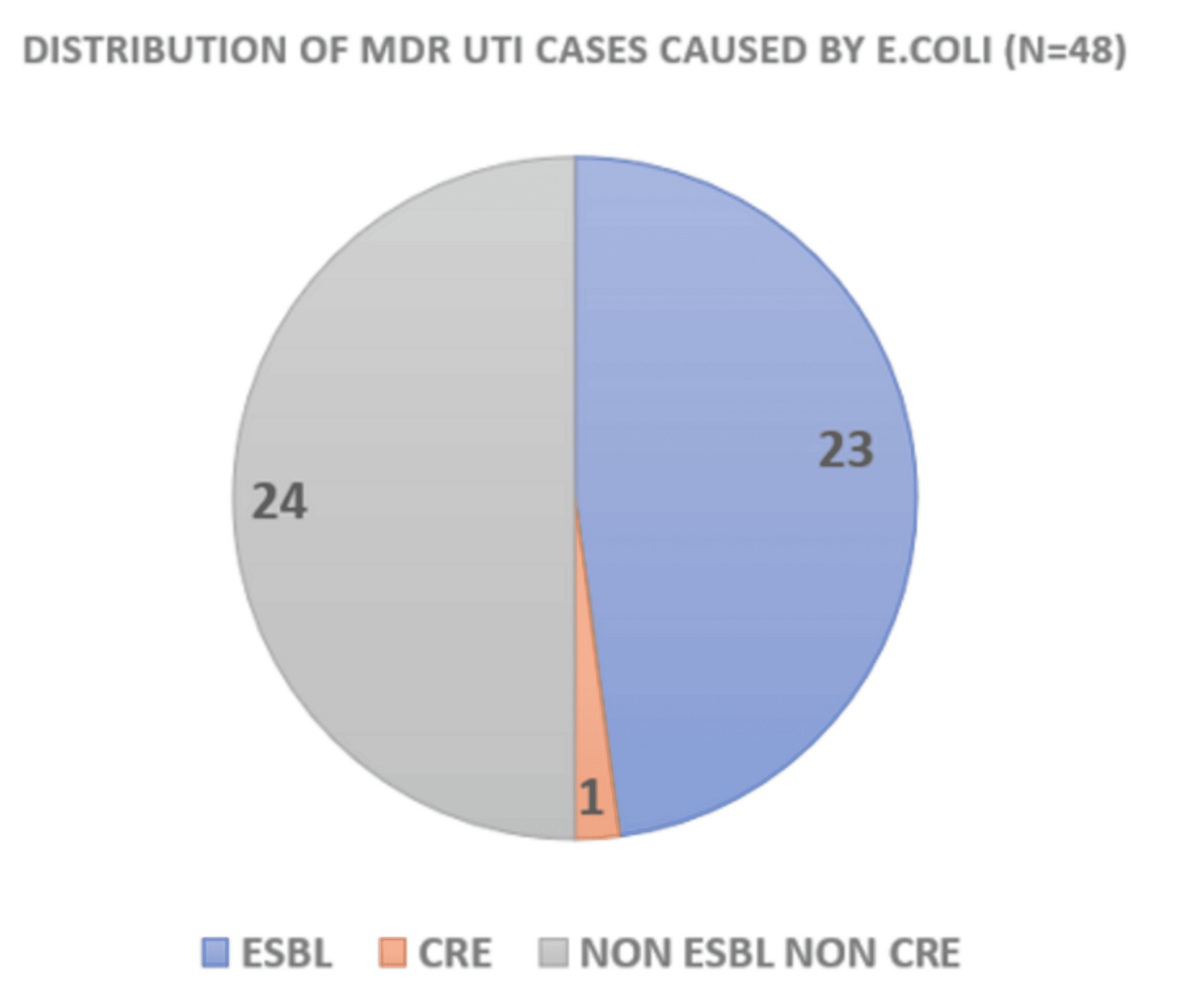
Phenotypic distribution of Escherichia coli isolates in MDR UTI cases (N = 48) MDR UTI: multidrug-resistant urinary tract infection; *E. coli*: *Escherichia coli*; ESBL: non-extended-spectrum beta-lactamase; CRE: carbapenem-resistant *Enterobacterales*

**Figure 6:**
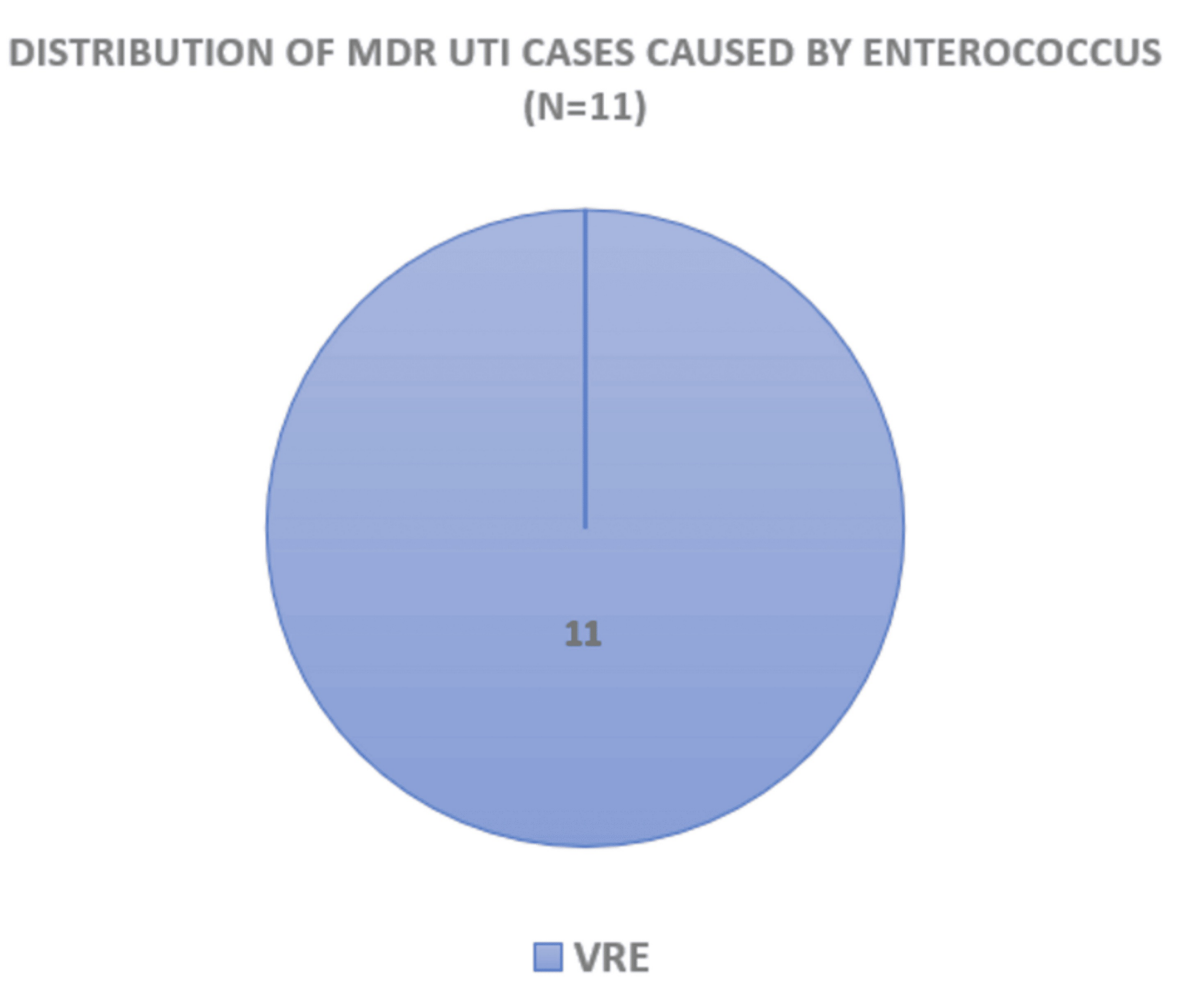
Phenotypic distribution of Enterococcus isolates in MDR UTI cases (N = 11) MDR UTI: multidrug-resistant urinary tract infection; VRE: vancomycin-resistant enterococci

**Figure 7:**
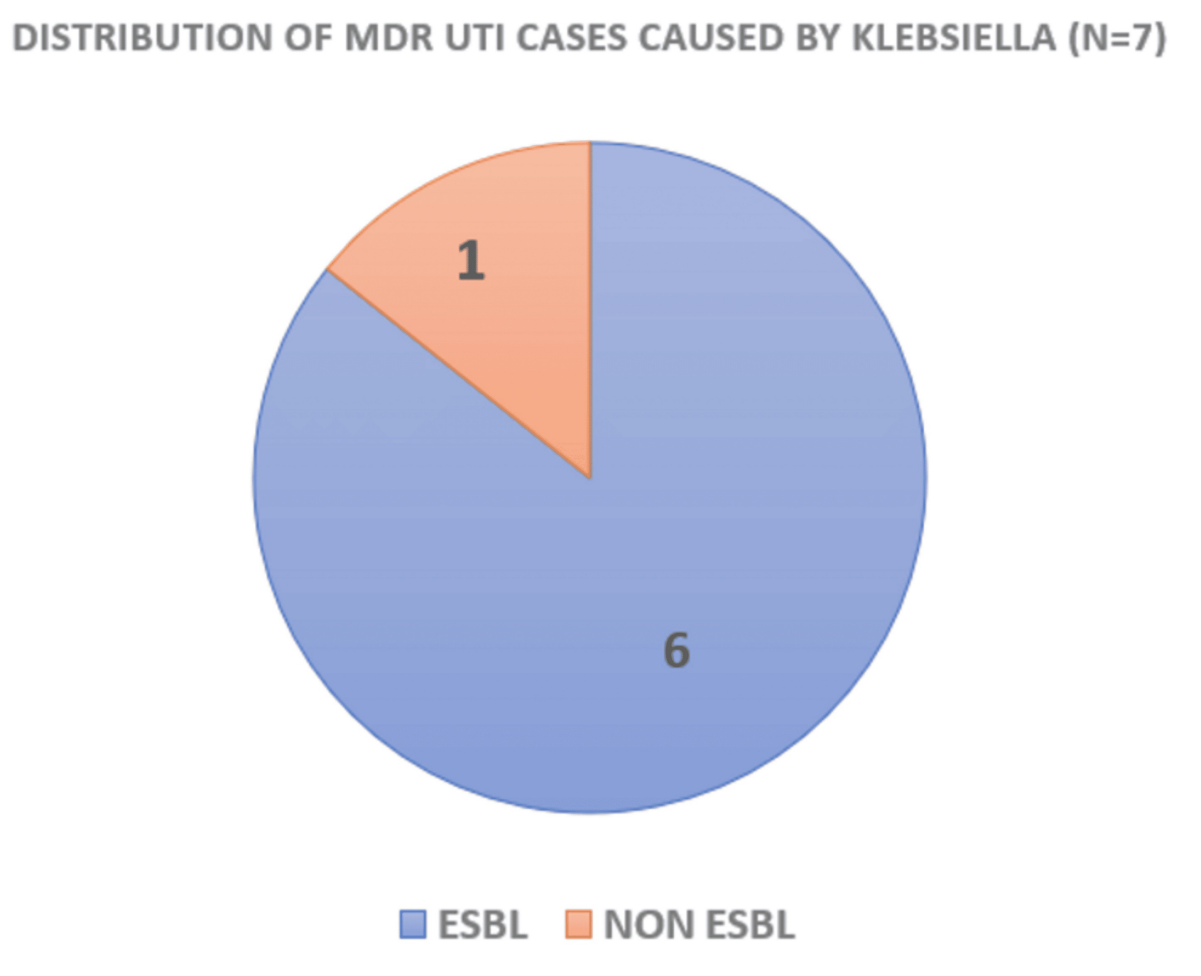
Phenotypic distribution of Klebsiella pneumoniae isolates in MDR UTI cases (N = 7) MDR UTI: multidrug-resistant urinary tract infection; ESBL: non-extended-spectrum beta-lactamase

**Figure 8:**
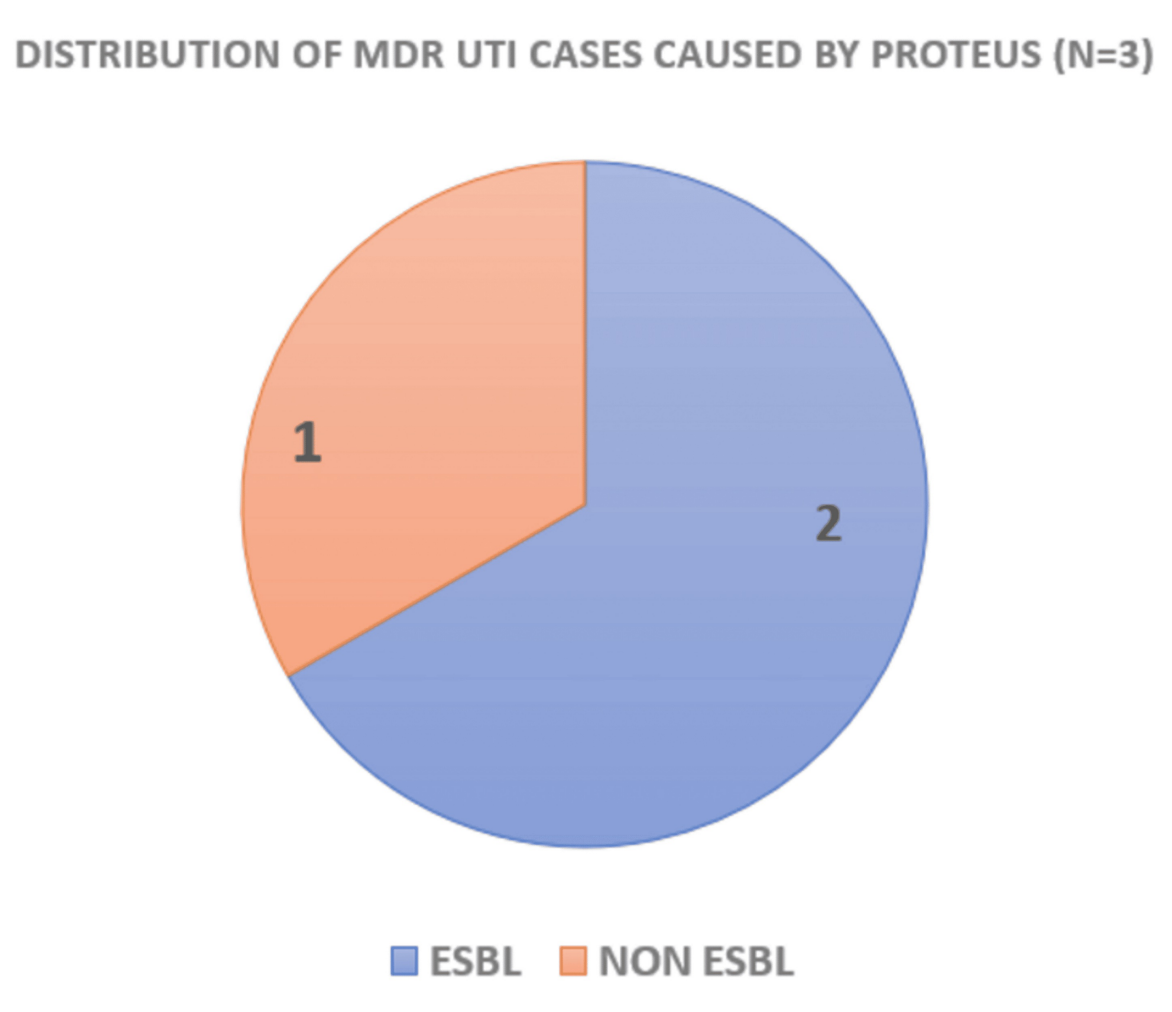
Phenotypic distribution of Proteus isolates in MDR UTI cases (N = 3) MDR UTI: multidrug-resistant urinary tract infection; ESBL: non-extended-spectrum beta-lactamase

The MDR uropathogens isolated from urine samples included *Escherichia coli*, *Enterococcus*, *Klebsiella pneumoniae*, *Proteus*, and *Pseudomonas *(Figure 4). Of 70 samples, 48 samples (68.57%) showed growth of *Escherichia coli*. Of these 48, 24 (50%) were non-extended-spectrum beta-lactamase (ESBL)-producing and non-carbapenem-resistant *Enterobacterales *(CRE) strains, 23 were ESBL-producing strains (47.91%), and one was a CRE strain (2.08%) (Figure 5). *Enterococcus *was detected in 11 of 70 samples (15.71%), all of which were vancomycin-resistant (100%) (Figure 6). *Klebsiella *was found in seven of 70 samples (10%), of which six produced ESBL (85.71%), and one did not (14.28%) (Figure 7). *Proteus *was present in three of 70 samples (4.28%), including two ESBL producers (66.66%) and one non-ESBL strain (33.33%) (Figure 8). *Pseudomonas *was identified in one of 70 samples (1.42%).

**Figure 9:**
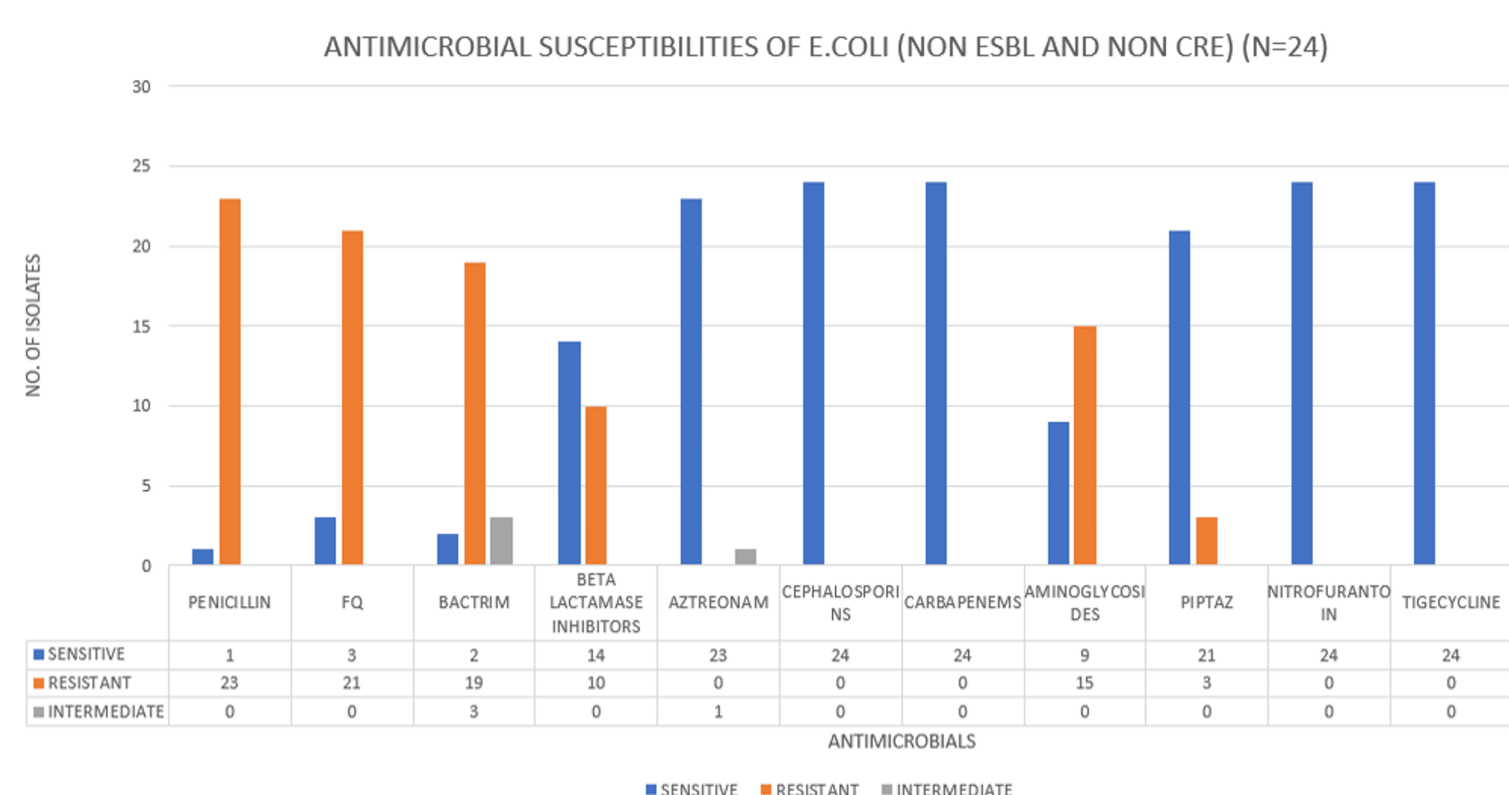
Antimicrobial susceptibility pattern of non-ESBL and non-CRE Escherichia coli isolates causing MDR UTI (N = 24) MDR UTI: multidrug-resistant urinary tract infection; *E. coli*: *Escherichia coli*; ESBL: non-extended-spectrum beta-lactamase; CRE: carbapenem-resistant *Enterobacterales*; FQ: fluoroquinolones; PIPTAZ: piperacillin-tazobactam

Of the 24 tested non-ESBL and non-CRE *Escherichia coli* isolates, all were susceptible to cephalosporins, carbapenems, nitrofurantoin, and tigecycline. Additionally, 23 of 24 isolates (95.83%) were susceptible to aztreonam and 21 (87.5%) to piperacillin-tazobactam. A moderate susceptibility rate was noted for beta-lactamase inhibitor combinations at 58.33% (14 isolates) and for aminoglycosides at 37.5% (nine isolates). A very low susceptibility rate was observed with penicillin at 4.16% (one isolate), fluoroquinolones at 12.5% (three isolates), and TMP-SMZ at 8.33% (two isolates). Resistance was most common to penicillin (23, 95.83%), followed by fluoroquinolones (21, 87.5%), TMP-SMZ (19, 79.16%), and aminoglycosides (15, 62.5%). Only minimal intermediate susceptibility was seen with TMP-SMZ (three, 12.5%) and aztreonam (one, 4.16%) (Figure 9).

**Figure 10:**
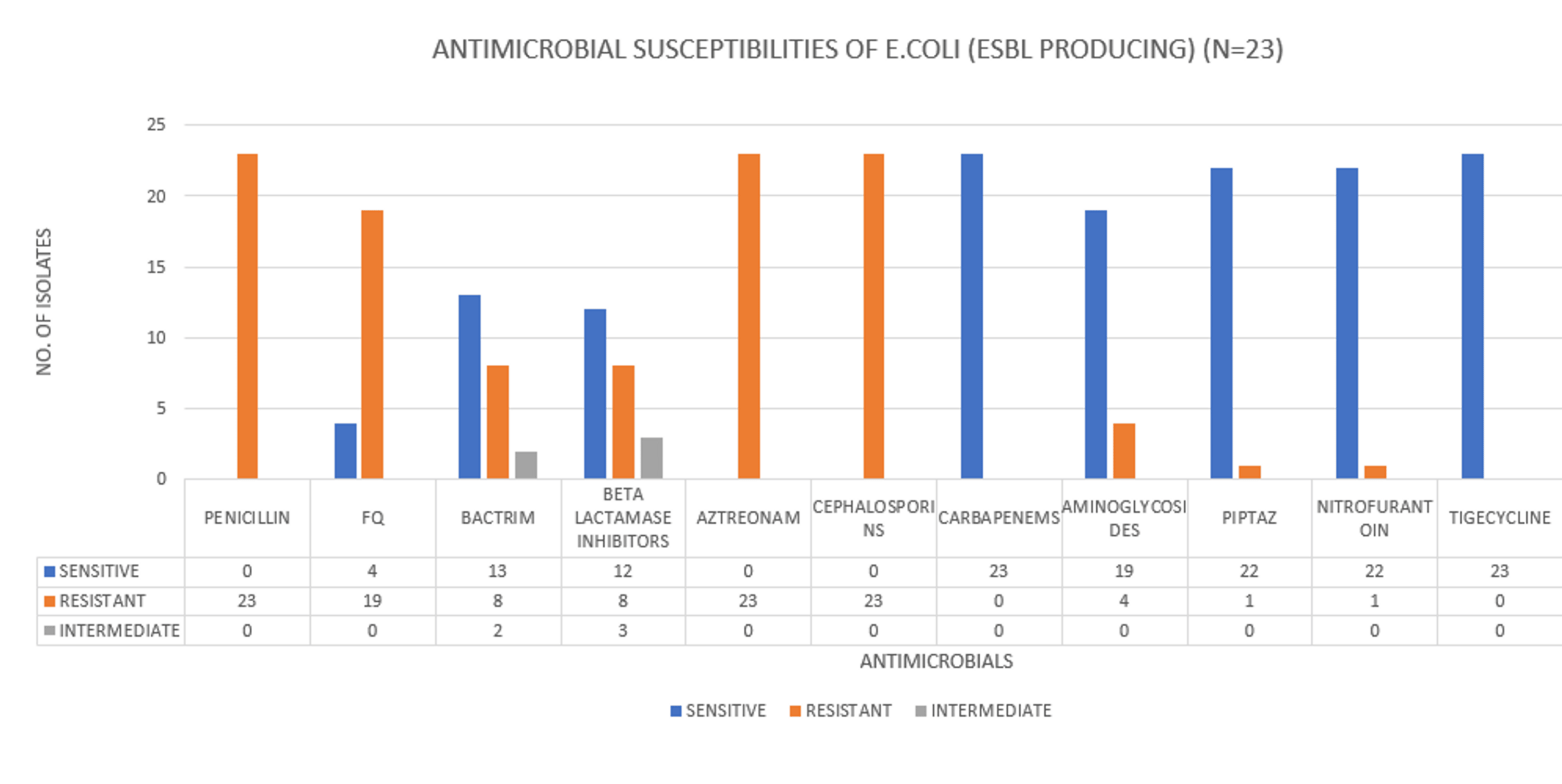
Antimicrobial susceptibility pattern of ESBL-producing Escherichia coli isolates causing MDR UTI (N = 23) MDR UTI: multidrug-resistant urinary tract infection; *E. coli*: *Escherichia coli*; ESBL: non-extended-spectrum beta-lactamase; FQ: fluoroquinolones; PIPTAZ: piperacillin-tazobactam

Among the 23 ESBL-producing *Escherichia coli* isolates tested, all were susceptible to carbapenems and tigecycline. A high susceptibility rate was also noted for piperacillin-tazobactam and nitrofurantoin (22 isolates, 95.65%), followed by aminoglycosides (19 isolates, 82.6%). Moderate susceptibility rates were observed to TMP-SMZ (13 isolates, 56.52%) and beta-lactamase inhibitor combinations (12 isolates, 52.17%). In contrast, all isolates were resistant to penicillin, aztreonam, and cephalosporins. A high rate of resistance was also observed to fluoroquinolones (19 isolates, 82.6%). Intermediate susceptibility was minimal, observed only with TMP-SMZ (two isolates, 8.69%) and beta-lactamase inhibitor combinations (three isolates, 13.04%) (Figure 10).

**Figure 11:**
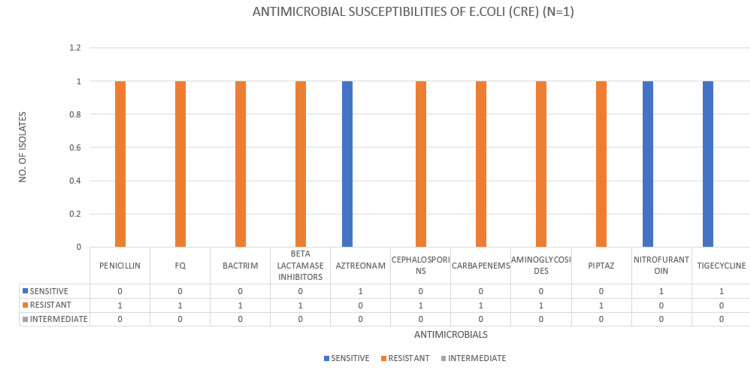
Antimicrobial susceptibility pattern of CRE E. coli isolate causing MDR UTI (N = 1) MDR UTI: multidrug-resistant urinary tract infection; *E. coli*: *Escherichia coli*; CRE: carbapenem-resistant *Enterobacterales*; FQ: fluoroquinolones; PIPTAZ: piperacillin-tazobactam

The single CRE *Escherichia coli* isolate was only susceptible to aztreonam, nitrofurantoin, and tigecycline. It was completely resistant to penicillin, fluoroquinolones, TMP-SMZ, beta-lactamase inhibitors, cephalosporins, carbapenems, aminoglycosides, and piperacillin-tazobactam. No antimicrobials tested showed intermediate susceptibility (Figure 11). 

**Figure 12:**
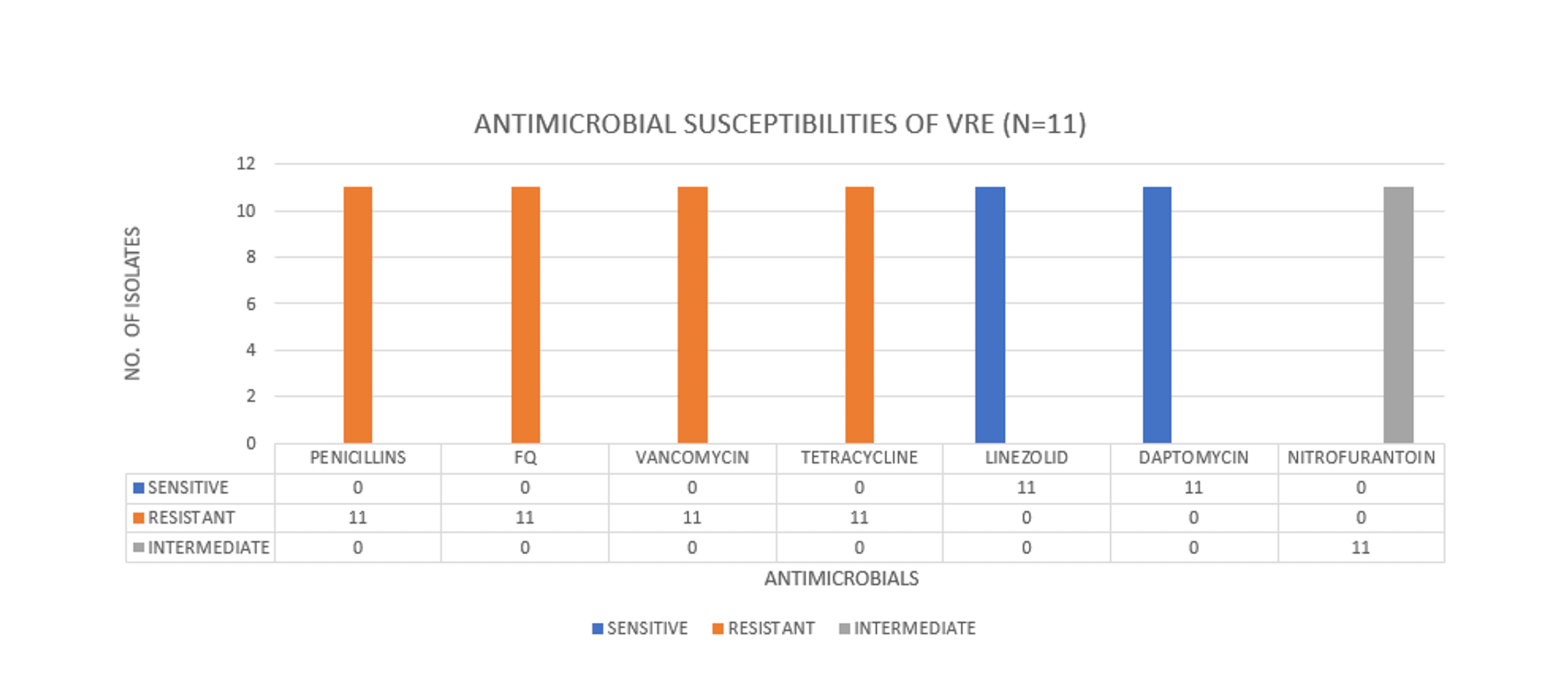
Antimicrobial susceptibility pattern of VRE isolates (N = 11) VRE: vancomycin-resistant enterococci; FQ: fluoroquinolones

Among the 11 vancomycin-resistant *Enterococcus *(VRE) isolates tested, all were resistant to penicillins, fluoroquinolones, vancomycin, and tetracycline. In contrast, all isolates were fully susceptible to linezolid and daptomycin. Nitrofurantoin demonstrated intermediate susceptibility across all isolates, with none categorised as either sensitive or resistant (Figure 12). 

**Figure 13:**
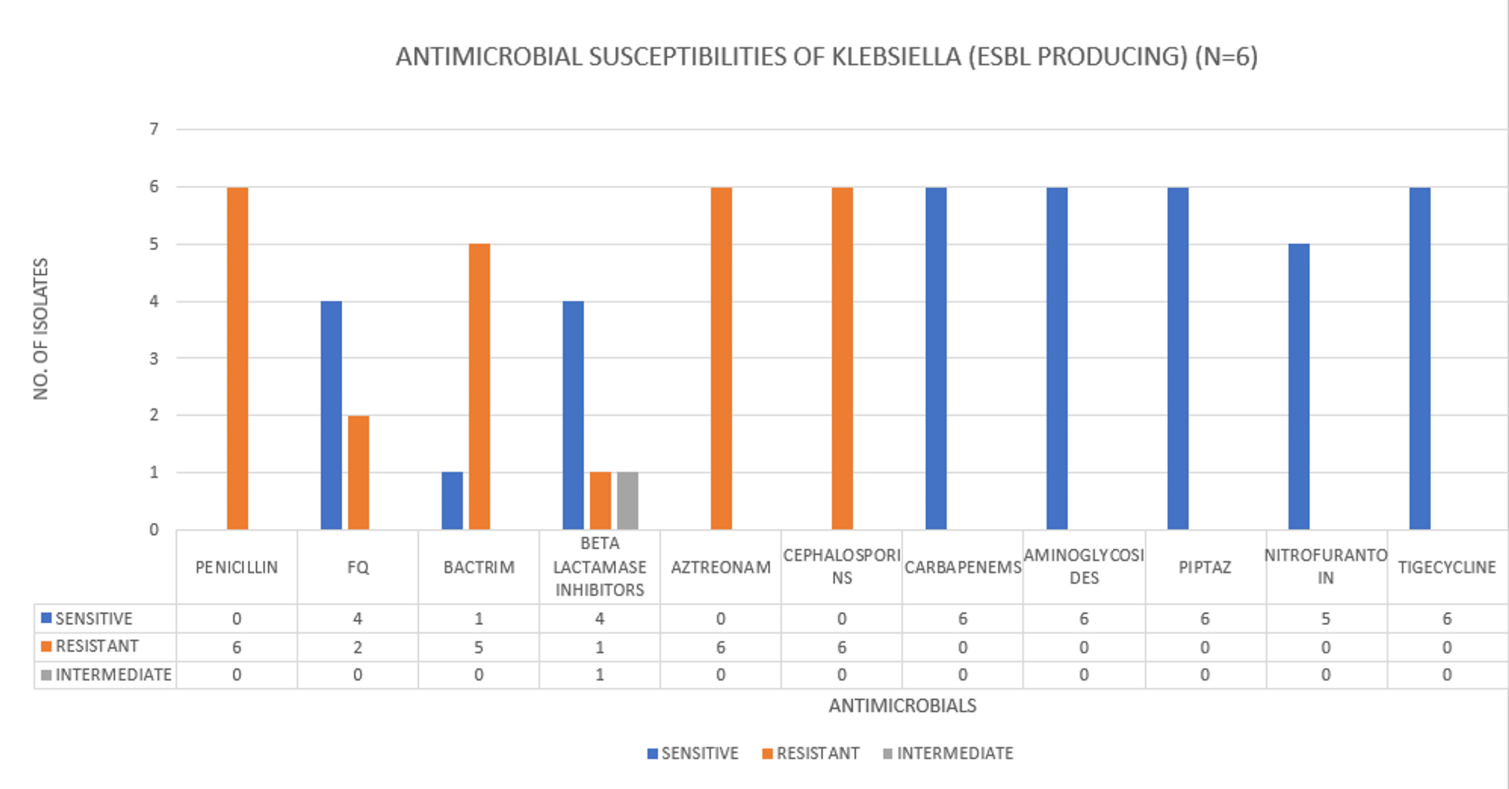
Antimicrobial susceptibility pattern of ESBL-producing Klebsiella isolates causing MDR UTI (N = 6) MDR UTI: multidrug-resistant urinary tract infection; ESBL: non-extended-spectrum beta-lactamase; FQ: fluoroquinolones; PIPTAZ: piperacillin-tazobactam

Among the six ESBL-producing *Klebsiella pneumoniae* isolates tested, all were susceptible to carbapenems, aminoglycosides, piperacillin-tazobactam, and tigecycline. High susceptibility was also noted for nitrofurantoin (five isolates, 83.3%), fluoroquinolones (four isolates, 66.7%), and beta-lactamase inhibitor combinations (four isolates, 66.7%). In contrast, all isolates were completely resistant to penicillin, aztreonam, and cephalosporins. High resistance was also observed to TMP-SMZ (five isolates, 83.3%). Intermediate susceptibility was minimal and observed only with beta-lactamase inhibitor combinations (one isolate, 16.7%) (Figure 13). 

**Figure 14:**
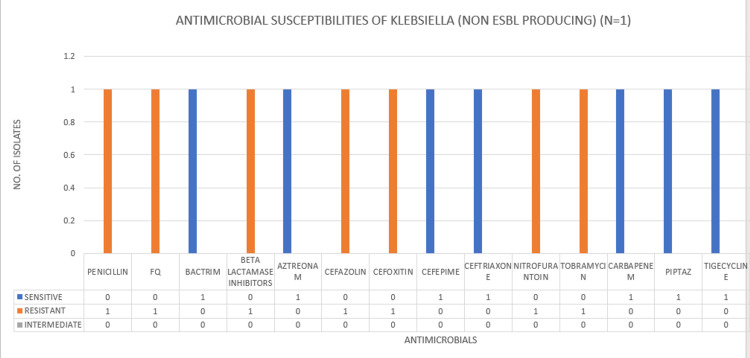
Antimicrobial susceptibility pattern of non-ESBL Klebsiella isolate causing MDR UTI (N = 1) MDR UTI: multidrug-resistant urinary tract infection; ESBL: non-extended-spectrum beta-lactamase; FQ: fluoroquinolones; PIPTAZ: piperacillin-tazobactam

The single non-ESBL-producing *Klebsiella pneumoniae* isolate demonstrated susceptibility to TMP-SMZ, aztreonam, cefepime, ceftriaxone, carbapenem, piperacillin-tazobactam, and tigecycline. In contrast, the isolate showed complete resistance to penicillin, fluoroquinolones, beta-lactamase inhibitor combinations, cefazolin, cefoxitin, nitrofurantoin, and tobramycin. No intermediate susceptibility was observed for any of the antimicrobials tested (Figure 14).

**Figure 15:**
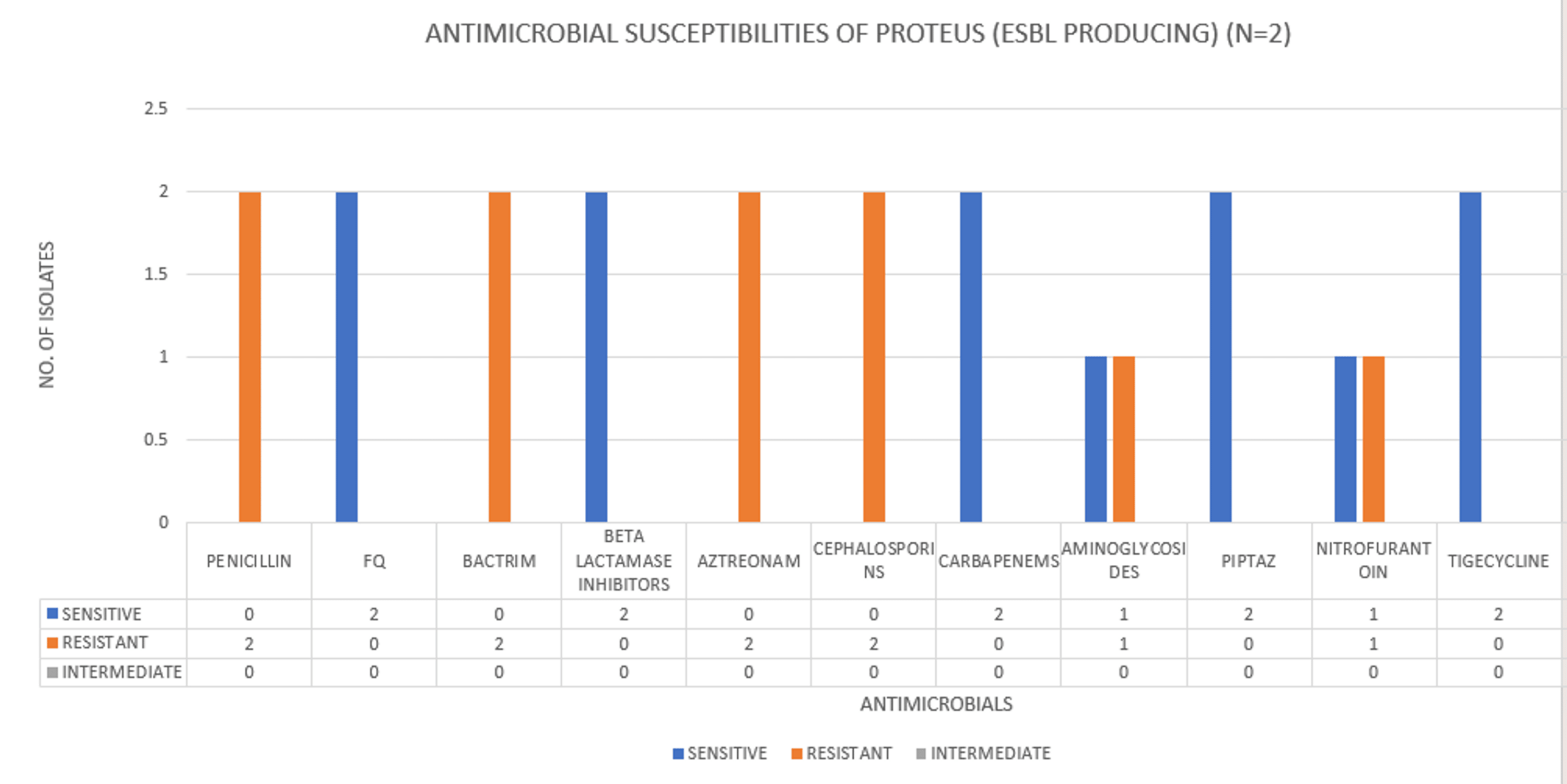
Antimicrobial susceptibility pattern of ESBL-producing Proteus isolates causing MDR UTI (N = 2) MDR UTI: multidrug-resistant urinary tract infection; ESBL: non-extended-spectrum beta-lactamase; FQ: fluoroquinolones; PIPTAZ: piperacillin-tazobactam

Among the two ESBL-producing *Proteus *isolates tested, complete susceptibility was observed to fluoroquinolones, beta-lactamase inhibitor combinations, carbapenems, piperacillin-tazobactam, and tigecycline. Moderate susceptibility was observed to aminoglycosides and nitrofurantoin (one isolate; 50%). In contrast, all isolates were completely resistant to penicillin, TMP-SMZ, aztreonam, and cephalosporins. Aminoglycosides and nitrofurantoin also demonstrated 50% resistance (one isolate each). No intermediate susceptibility was observed for any of the antimicrobials tested (Figure 15).

**Figure 16:**
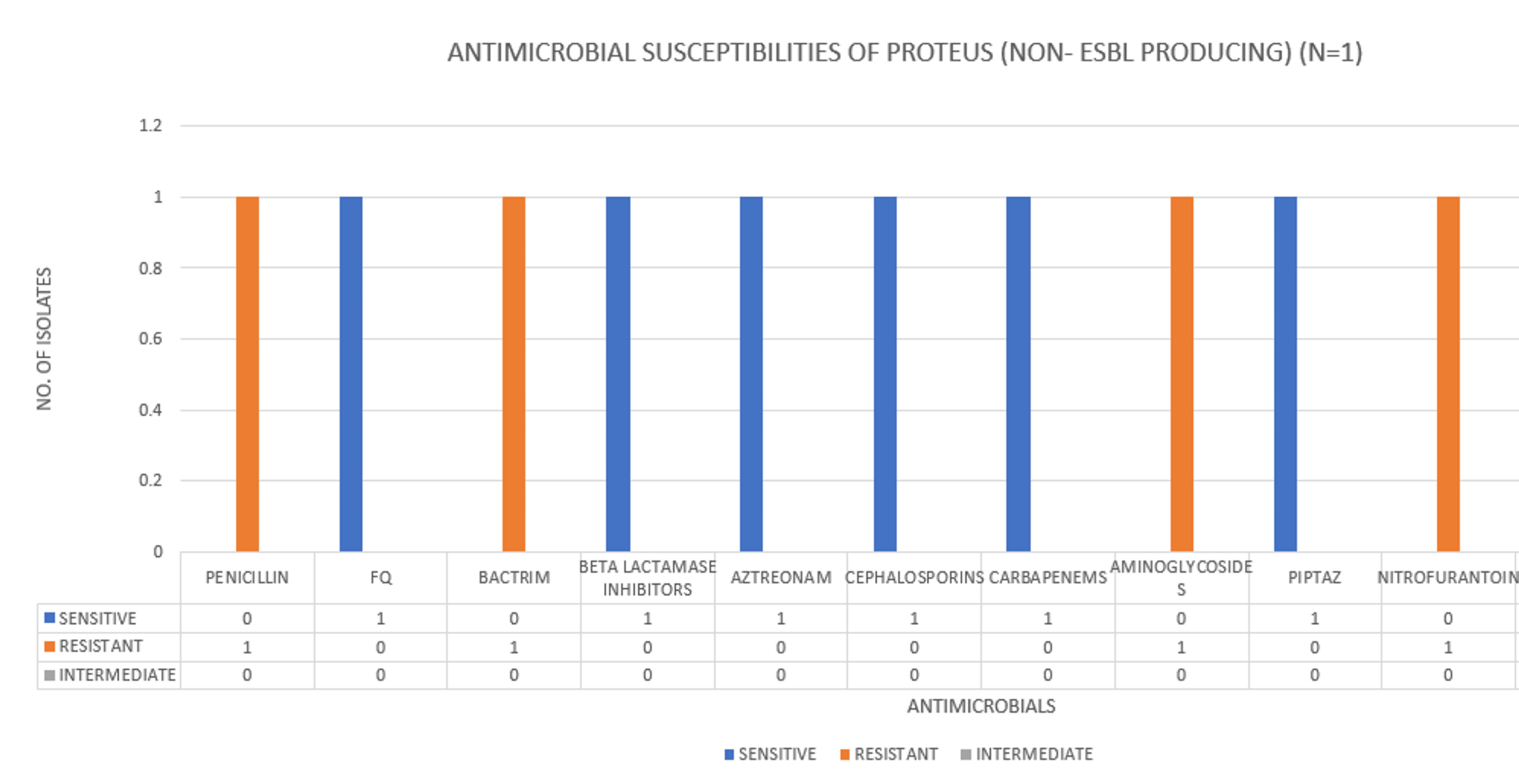
Antimicrobial susceptibility pattern of non-ESBL Proteus isolate causing MDR UTI (N = 1) MDR UTI: multidrug-resistant urinary tract infection; ESBL: non-extended-spectrum beta-lactamase; FQ: fluoroquinolones; PIPTAZ: piperacillin-tazobactam

The single non-ESBL-producing *Proteus *isolate demonstrated complete susceptibility to fluoroquinolones, beta-lactamase inhibitor combinations, aztreonam, cephalosporins, carbapenems, and piperacillin-tazobactam. In contrast, the isolate showed complete resistance to penicillin, TMP-SMZ, aminoglycosides, and nitrofurantoin. No intermediate susceptibility was observed for any of the antimicrobials tested (Figure 16).

**Figure 17:**
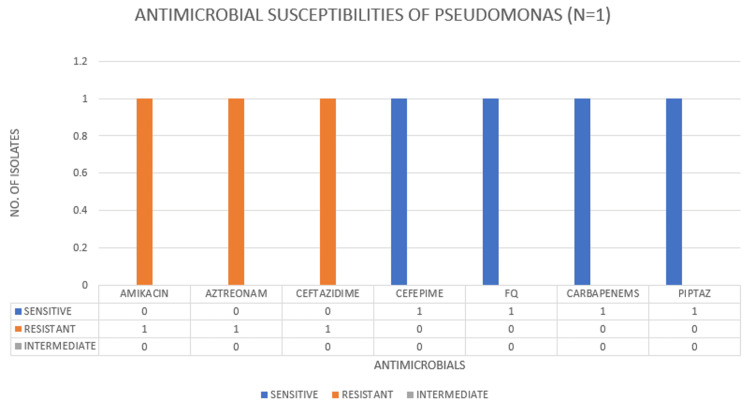
Antimicrobial susceptibility pattern of Pseudomonas isolate causing MDR UTI (N = 1) MDR UTI: multidrug-resistant urinary tract infection; FQ: fluoroquinolones; PIPTAZ: piperacillin-tazobactam

The single *Pseudomonas *isolate was susceptible to cefepime, fluoroquinolones, carbapenems, and piperacillin-tazobactam. In contrast, the isolate showed complete resistance to amikacin, aztreonam, and ceftazidime. No intermediate susceptibility was observed for any of the antimicrobials tested (Figure 17).

## Discussion

Our study revealed that 24.47% (70/286) of patients with symptomatic UTIs harbored MDR organisms, emphasizing the increasing challenge of antimicrobial resistance among urinary pathogens. This rate aligns with existing studies reporting MDR prevalence between 24.5% and 41.4% in hospitalized UTI patients [17,18]. The slightly lower rate in our group might be due to differences in patient demographics, local resistance patterns, or the exclusion of asymptomatic bacteriuria and colonization. Most MDR infections were observed in females (56/70; 80%). Although female sex is a known risk factor for UTI occurrence and recurrence, its status as an independent predictor of MDR UTIs remains debatable, with studies showing conflicting outcomes depending on the population and setting. Some research links female sex to higher MDR UTI risk [19-21], while others suggest males have a greater risk [22-25]. These findings imply that the link between female sex and MDR UTIs is largely influenced by different antibiotic exposure patterns, due to the higher frequency and recurrence of UTIs in women, rather than innate biological susceptibility. Recognizing this distinction is crucial. Antibiotic stewardship efforts focused on women with recurrent UTIs, such as non-antibiotic prophylactics and targeted empiric therapy based on local resistance data, could significantly reduce the overall burden of MDR UTIs. 

In our cohort, previous antibiotic use within the last six months was recorded in 42 of 70 patients (60%), making it the most common clinical feature. This aligns with a systematic review by Tenney et al. [26], which found prior antibiotic use to be the most frequently linked characteristic with MDR UTIs, present in 16 of 20 studies analyzing this factor. Malcolm et al. [27] further quantified this at the population level using data from over 40,000 community urine isolates in Scotland, showing that taking four or more different antibiotics in the past six months increased the risk of MDR nearly sevenfold. Notably, cephalosporins were the most frequently used antibiotics before infection (35/42 patients; 83.33%), followed by fluoroquinolones (14/42 patients; 33.33%). The common prior use of cephalosporins is especially relevant given the high prevalence of ESBL-producing organisms in our study, as cephalosporin exposure is a key driver of ESBL development in pathogens [28].

Previous hospitalization within the last 12 months was recorded for 41 out of 70 patients (58.57%), making it the second most common clinical feature in our cohort. This matches the findings of Onorato et al. [17], who found that hospital admission within the past 90 days is an independent predictor of MDR pathogen detection. Macesic et al. [29], in a thorough Lancet review of MDR Gram-negative infections, listed previous hospitalization as a consistent clinical characteristic across all resistance categories. The IDSA 2025 guideline [30] also noted that healthcare exposure to a hospital or nursing home within the past three months is a predictor of resistant uropathogens, although the link may be mediated by other factors, such as prior antibiotic use, comorbidities, and indwelling devices.

Comorbid conditions such as type 2 diabetes mellitus, chronic kidney disease, immunosuppression, and neurogenic bladder were found in 31 out of 70 patients (44.28%). The link between diabetes and MDR UTIs is well-documented. A systematic review and meta-analysis by Carrillo-Larco et al. [31] showed that patients with type 2 diabetes are twice as likely to develop antibiotic-resistant UTIs.

Catheterization within the past 12 months was recorded in 15 of 70 patients (21.42%), while 29 patients (41.42%) had a history of UTIs in the same period. Both factors are well-known links to MDR UTIs [26]. 

Twenty-two out of 70 patients (31.42%) had documented residence in assisted living facilities, nursing homes, or group homes. Long-term care facility (LTCF) residence is a consistently identified factor associated with MDR UTIs in research. Garcia-Bustos et al. [32] identified it as the strongest independent predictor of MDR infection. Rosello et al. [33], in a population-based study of over 650,000 urine samples in England, found LTCF residents had more than twice the rate of *Escherichia coli* and *Klebsiella *UTIs and over four times the rate of antibiotic-resistant bacteria-caused UTIs compared to community-dwelling elderly people. Eure et al. [34], analyzing data from 243 US nursing homes reported to the National Healthcare Safety Network, observed that 36% of UTIs involved resistant pathogens, with fluoroquinolone-resistant *Escherichia coli* reaching 50% and vancomycin-resistant *Enterococcus faecium *reaching 60%. The high prevalence of MDR organisms in LTCFs is linked to multiple factors, including frequent antibiotic use, indwelling devices, close living conditions promoting horizontal transmission, and a population with significant comorbidities.

*Escherichia coli* was the predominant MDR uropathogen in our study, accounting for 48 of 70 isolates (68.57%). This is consistent with the global literature, which consistently identifies *Escherichia coli *as the most common cause of UTIs, responsible for 75%-90% of all cases [35]. Among the 48 *Escherichia coli* isolates, 23 (47.91%) were ESBL-producing, and one (2.08%) was carbapenem-resistant. The ESBL prevalence in our *Escherichia coli* isolates is notably higher than the 15.7% national prevalence reported by Critchley et al. [36] in a US-wide survey and the 17.2% prevalence among hospitalized ED patients reported by Talan et al. [37], but is consistent with the rising trend documented by Kaye et al. [38], who demonstrated a relative average yearly increase of 7.7% in ESBL-positive *Escherichia coli* isolates among US female outpatients from 2011 to 2019. The IDSA has noted that the incidence of ESBL-producing *Enterobacterales *in the United States increased by 53% from 2012 to 2017, driven largely by community-acquired infections [39]. Of the 70 isolates, *Klebsiella *species accounted for seven isolates (10%), of which six (85.71%) were ESBL-producing. This high ESBL rate among *Klebsiella *is consistent with the findings of Gomila et al. [40], who reported a 54.2% MDR rate in *Klebsiella* pneumoniae, the highest among all uropathogens studied, in a multicenter European cohort. *Proteus *species accounted for three of 70 isolates (4.28%), with two being ESBL-producing, consistent with the recognition of *Proteus mirabilis* as one of the organisms in which ESBL genes are most prevalent [41].

A particularly notable finding was that all 11 *Enterococcus *isolates of 70 samples in the MDR UTI cohort (15.71%) were vancomycin-resistant. This is a concerning proportion, though it must be interpreted in the context of the study's focus on MDR organisms. Enterococci are recognized as important uropathogens, particularly in healthcare-associated settings. Eure et al. reported that although *Enterococcus faecium* accounted for less than 5% of UTI pathogens in US nursing homes, it had the highest rate of vancomycin resistance, at 60% [34]. Heintz et al. have emphasized the importance of differentiating VRE colonization from true infection, as unnecessary treatment of colonized patients contributes to the selection of further resistance [42]. The high VRE rate in our cohort likely reflects the healthcare-associated nature of many of these infections, given that 58.71% of patients had prior hospitalization and 31.42% were LTCF residents.

A single *Pseudomonas aeruginosa* isolate (1/70; 1.42%) was identified, consistent with its relatively lower prevalence in UTIs compared with other sites of infection, though it remains an important pathogen in complicated and catheter-associated UTIs.

This study reveals several key patterns in susceptibility data that impact empiric prescribing and antibiotic stewardship. Carbapenems and tigecycline show a high degree of effectiveness against all tested gram-negative organisms, confirming their reliability for serious MDR UTIs. Nonetheless, the IDSA recommends carbapenem-sparing approaches when possible, favoring oral TMP-SMZ or fluoroquinolones (if susceptible) as step-down therapies for ESBL-*Enterobacterales pyelonephritis* and complicated UTIs to help preserve carbapenem efficacy for future resistant infections [30]. It is also important to note that although tigecycline demonstrated excellent in vitro activity, its limited urinary excretion restricts its clinical utility in UTIs. Nitrofurantoin maintains excellent activity against both non-ESBL and ESBL-producing *Escherichia coli*, supporting its role as a first-line treatment for uncomplicated cystitis according to the current guidelines [30]. However, its lack of activity against Proteus species and limited efficacy against *Klebsiella *and *Pseudomonas *highlight the importance of culture-guided therapy. The high rates of resistance to fluoroquinolones and TMP-SMZ across all *Escherichia coli* phenotypes raise concerns about the empiric use in populations with high MDR prevalence. The IDSA 2025 guidelines note that about 30% of *Enterobacterales *isolates nationwide are fluoroquinolone-resistant, and our data suggest this rate is likely higher in MDR-enriched populations [30]. The 100% susceptibility of VRE to linezolid and daptomycin reassures that effective treatments remain available for enterococcal MDR UTI, though these should be reserved for confirmed infections rather than colonization, as Heintz et al. emphasize [42].

These findings collectively underscore the importance of obtaining urine cultures and susceptibility testing before initiating or modifying antibiotic therapy, particularly in patients at risk of MDR infection. Empiric regimens should be guided by local antibiograms and individual patient characteristics, with de-escalation to the narrowest effective agent once susceptibility data are available.

Limitations of the study

While this study offers valuable insights into the prevalence and antibiotic susceptibility patterns of MDR UTIs, several limitations should be acknowledged. It was conducted at a single community hospital in Sleepy Hollow, NY, USA. Since susceptibility patterns vary widely by geography and healthcare settings, the findings may not apply to other regions or larger tertiary centers. As a retrospective study using de-identified secondary data, researchers relied on existing lab and hospital records, limiting control over confounding variables and preventing verification of clinical details beyond documentation. The data collection spanned only four months (February to May 2026), which may not reflect seasonal infection trends or long-term resistance shifts. All samples were from inpatients, excluding outpatients with community-acquired infections who did not need hospitalization, likely leading to an overestimation of resistance prevalence. Although 70 MDR cases were identified, some uropathogens had very few isolates, such as only one Pseudomonas and one CRE *Escherichia coli*, preventing statistically meaningful analysis of their resistance profiles. Limited availability of certain antibiotic discs restricted uniform susceptibility testing across all isolates, potentially reducing data comprehensiveness. Due to the study's descriptive, local surveillance nature, small sample sizes for some bacteria, and reliance on secondary data, no formal inferential statistical analysis was conducted. Results are instead presented as frequencies and percentages, emphasizing immediate clinical and resistance trends within this institution.

## Conclusions

MDR UTIs pose a significant and increasing challenge in hospitalized patients. Overall, this study underscores the clinical significance of MDR UTIs and highlights the importance of antimicrobial stewardship, continuous surveillance of resistance patterns, and evidence-based therapeutic strategies to improve patient care. A high prevalence of ESBL-producing organisms and VRE amongst the MDR cohort reflects the growing problem of antimicrobial resistance in healthcare-associated infections. Factors such as previous antibiotic use, prior hospitalization, recurrent UTIs, comorbidities, catheterization, and residence in long-term care facilities were identified as important contributors to MDR UTIs. The antimicrobial susceptibility patterns showed that carbapenems, tigecycline, linezolid, and daptomycin largely retained activity against most MDR isolates, while resistance rates remained high for penicillins, fluoroquinolones, cephalosporins, and TMP-SMX.

These results highlight the importance of routine urine cultures and antimicrobial susceptibility testing, especially in high-risk patients. Empiric treatment should be guided by local antibiograms and individual patient factors, with quick de-escalation to the narrowest effective therapy when possible. Enhancing antimicrobial stewardship and implementing targeted infection control strategies are vital for reducing the emergence and spread of MDR uropathogens and maintaining the efficacy of existing antibiotics. Additionally, regional surveillance is necessary to monitor changes in antibiotic susceptibility patterns.
